# Chinese Systemic Lupus Erythematosus Treatment and Research Group Registry VI: Effect of Cigarette Smoking on the Clinical Phenotype of Chinese Patients with Systemic Lupus Erythematosus

**DOI:** 10.1371/journal.pone.0134451

**Published:** 2015-08-17

**Authors:** Dong Xu, Xin You, Zhengang Wang, Qingyu Zeng, Jianhua Xu, Lindi Jiang, Lu Gong, Fengqi Wu, Jieruo Gu, Yi Tao, Jinwei Chen, Jiuliang Zhao, Mengtao Li, Yan Zhao, Xiaofeng Zeng, CSTAR co-authors

**Affiliations:** 1 Department of Rheumatology, Peking Union Medical College Hospital, Peking Union Medical College & Chinese Academy of Medical Sciences; Key Laboratory of Rheumatology and Clinical Immunology, Ministry of Education; No.1 Shuaifuyuan, Beijing, 100730, China; 2 Department of Rheumatology, Beijing Tongren Hospital Affiliated to Capital Medical University, NO 1, Dong Jiao Min Xiang, Eastern District, Beijing, 100730, China; 3 Department of Rheumatology, the First Affiliated Hospital of Shantou University Medical College, No.57 ChangPingLu, Shantou, 515041, China; 4 Department of Rheumatology, the First Affiliated Hospital of Anhui Medical University, No.218 JiXiLu, Hefei, 230022, China; 5 Department of Rheumatology, Zhongshan Hospital Affiliated to Fudan University, No.180 FengLinLu, Shanghai, 200032, China; 6 Department of Rheumatology, the General Hospital of Tianjing Medical University, No.154 AnShanDao, Tianjing, 300052, China; 7 Department of Rheumatology, Capital Institute of Pediatrics, No.2 YaBaoLu, Beijing, 100020, China; 8 Department of Rheumatology, the Third Affiliated Hospital of Sun Yat-sen University, No.600 TianHeLu, Guangzhou, 510630, China; 9 Department of Rheumatology, the Second Affiliated Hospital of Guangzhou Medical College, No.107 YanJiangXiLu, Guangzhou, 510120, China; 10 Department of Rheumatology, the Second Xiangya Hospital of Central South University, No.139 RenMinZhongLu, Changsha, 410011, China; Penn State University, UNITED STATES

## Abstract

**Objectives:**

Our study aimed to investigate the effect of cigarette smoking on the clinical phenotype of patients registered in the Chinese Systemic Lupus Erythematosus (SLE) Treatment and Research (CSTAR) group registry database, the first online registry of Chinese patients with SLE.

**Methods:**

A prospective cross-sectional study of Chinese SLE patients was conducted using the CSTAR. Our case-control analysis was performed on age- and gender-matched subjects to explore the potential effect of cigarette smoking on the clinical manifestation of SLE.

**Results:**

Smokers comprised 8.9% (65/730) of patients, and the ratio of females/males was 19/46. Thirty-nine patients were current smokers, and 26 were ex-smokers. Data showed significant differences between smokers and nonsmokers in the following areas: nephropathy (58.5% vs. 39.2%; p = 0.003), microscopic hematuria (30.8% vs. 19.1%; p = 0.025), proteinuria (53.8% vs. 34.4%; p = 0.002), and SLE Disease Activity Index(DAI) scores (12.38±8.95 vs. 9.83±6.81; p = 0.028). After adjusting for age and gender, significant differences between smokers and nonsmokers were found with photosensitivity (35.9% vs. 18%; p = 0.006), nephropathy (59.4% vs. 39.8%; p = 0.011), and proteinuria (54.7% vs. 35.2%). Although smokers tended to have greater disease severity compared with nonsmokers (SLEDAI scores: 12.58±8.89 vs.10.5±7.09), the difference was not significant (p = 0.081).

**Conclusions:**

Cigarette smoking triggers the development and exacerbation of SLE, especially with respect to renal involvement. Chinese smokers with SLE should be advised to discontinue cigarette use.

## Introduction

Systemic lupus erythematosus (SLE) is an autoimmune disease characterized by multisystem involvement and generation of multiple autoantibodies. Although the etiology and pathogenesis of SLE are still unclear, exposure to environmental factors, such as infectious agents, drugs, occupational pollutants, and smoking, may play an important and complex role. Of these, smoking is one of the few potential causative factors that can be controlled by patient behavior. Cigarette smoke is known to affect the development and prognosis of many autoimmune diseases, especially rheumatoid arthritis. To date, the association between smoking and SLE has been controversial [1–6]. Most studies have shown that smoking is a risk factor for the development and severity of SLE in different races [2, 7–9]. Due to resource limitations, data on the relationship between SLE and smoking in the Chinese population has not been reported. Therefore, the Chinese SLE Treatment and Research group (CSTAR) developed the first online registry of Chinese patients with SLE, supported by the Chinese National Key Technology Research &Development Program. This registry has enabled the characterization of major clinical manifestations of SLE in Chinese patients [10] and provides the opportunity to prospectively study the effects of cigarette smoking on SLE clinical phenotypes in a representative cohort of Chinese patients.

## Methods

### Patient recruitment

This prospective cross-sectional analysis was based on the online CSTAR registry, which includes patients from 104 high-ranking rheumatology centers, covering 30 provinces in China. This study was approved by the Medical Ethics Committee of Peking Union Medical College Hospital (PUMCH), which was the lead research site; most centers accepted Ethics Committees(EC) from PUMCH as the leading site, some approved by their own EC, included Beijing Tongren Hospital, the General Hospital of TianJing Medical University, and the Second Affiliated Hospital of Guangzhou Medical College. Patients were registered only if they provided written informed consent. Patients with SLE were included only if they met the 1997 revised American College of Rheumatology criteria. Furthermore, patients were excluded if they presented with overlapping systemic sclerosis, rheumatoid arthritis, polymyositis, or other undifferentiated connective tissue diseases. This ongoing registry was launched in April 2009, and the cut-off for this study was February 2010.

### Data collection

All CSTAR centers used the same protocol-directed methods to provide uniform evaluations and patient data. All investigators received training on diagnostic confirmation of disease, evaluation of disease activity, as well as data input and quality control. Demographic data were also collected. Systemic manifestations (nervous system, vasculitis, arthritis, myositis, nephritis, rash, oral ulceration, pleuritis, pericarditis, and fever) were assessed using the SLE Disease Activity Index (SLEDAI), and all occurrences were classified according to SLEDAI definitions. Laboratory findings were also recorded, including leukocytopenia, thrombocytopenia, hypocomplementemia, and autoantibodies. Autoantibody levels were measured at local laboratories and included anti-double-stranded (ds) DNA, anti-Smith, anti-SSA/Ro, anti-SSB/La, anti-ribonucleoprotein (RNP) and anti-ribosomal RNP antibodies. SLE disease activity was evaluated in all patients by SLEDAI.

### Smoking definition and data collection

Patients who smoked at least one cigarette per day for three consecutive months were classified as smokers. Patients, who fulfilled the smoker criteria, but had given up smoking for at least 1 year prior to enrollment, were classified as ex-smokers. Patients who did not fulfill the criteria of smokers, were classified as nonsmokers[11]. Because history of smoking was not mandatory in the CSTAR, information regarding smoking behavior was obtainable in only 730 patients of 2104 who registered. The primary data of smoker in SLE patients are indicated in S1 Table.

### Control cases

For all subjects belonging to the smokers group, further analyses were performed to eliminate the confounding factors of gender and age. Gender and age were matched according to a 1:2 ratio for the recruitment of controls in CSTAR.

### Statistical analysis

The Statistical Package for the Social Sciences (SPSS) version 13.0 software (SPSS Inc, Chicago, IL, USA) was used for data processing and analysis. Variables were described using counts and/or percentages or medians and ranges. Gender and age were matched according to a 1:2 ratio, and smokers were compared to nonsmokers in a case-control study. Chi-squared and Fisher’s exact tests were used to compare categorical data, and independent sample Student’s *t*-tests were used to compare quantitative data between the groups. P-values <0.05 were considered to be statistically significant.

## Results

### Demographics

Out of 730 patients, 65 (8.9%) were either current or past smokers. Among these 65 patients, 19 were female, including 13 current and six ex-smokers, and 46 were male, including 26 current and 20 ex-smokers. Although SLE is a predominantly female disease, males are much more likely to smoke than females in the Chinese population. Thus, the ratio of female: male smokers in our SLE cohort was near 1:2.

### Clinical and laboratory findings

Compared to nonsmokers, there were more male smokers (46/65 vs.27/665), as well as more smokers patients with nephropathy (58.5% vs. 34.4%), microscopic hematuria (30.8% vs. 19.1%), proteinuria (53.8% vs. 34.4%), and highly active disease (SLEDAI scores: 12.38 ± 8.95 vs. 9.83 ± 6.81) (p<0.05). There was not a significant difference in autoantibodies between smokers and nonsmokers; however, anti-dsDNA positivity was slightly higher in smokers (p = 0.655; Tables 1 and 2).

**Table 1:** Comparison of clinical manifestations between smokers and nonsmokers in SLE patients.

	smokers(N = 65)	Nonsmokers(N = 665)	P value
Gender(F/M)	19/46	638/27	*<0*.*001*
Fever	21.5	20.5	0.836
Rash	43.1	36.8	0.321
Alopecia	26.2	25.3	0.875
Photosensitivity	35.4	26.6	0.130
Oral ulcers	18.5	11.1	0.080
Arthritis	47.7	53.5	0.368
Myositis	6.2	2.4	0.094
Pleuritis	16.9	9.6	0.064
Pericarditis	12.3	12.2	0.976
Vasculitis	12.3	6.6	0.089
Nephropathy	58.5	39.2	0.003
Renal cast	3.1	3.3	1.000
Microscopic hematuria	30.8	19.1	0.025
Proteinuria	53.8	34.4	0.002
Sterile pyuria	3.1	4.1	1.000
Nervous system	7.7	5.1	0.381
Hematological abnormalities	58.5	58.6	0.977

F, female; M, male

**Table 2:** Comparison of laboratory findings and SLEDAI scores between smokers and nonsmoker SLE patients.

	smokers(N = 65)	Nonsmokers(N = 665)	P value
Hypocomplementemia	60.0	69.6	0.110
ANA positivity	95.4	91.4	0.348
Anti dsDNA antibody positivity	55.4	52.5	0.655
Anti Sm antibody positivity	10.8	7.8	0.405
Anti RNP antibody positivity	16.9	11	0.152
Anti SSA antibody positivity	23.1	19.4	0.477
Anti SSB antibody positivity	16.9	11.7	0.222
Anti rRNP antibody positivity	26.9	17.9	0.257
APL antibody positivity	40.7	46.2	0.584
SLEDAI score	12.38±8.95	9.83±6.81	0.*028*

SLE: systemic lupus erythematosus; SLEDAI: SLE disease activity index; ANA: antinuclear antibody; Sm: Smith; RNP: ribonucleoprotein; rRNP: ribosomal RNP; APL: antiphospholipid.

### Case-control study

Because one male smoker was 78-years-old and no matching patients could be found, this patient was excluded from the case-control study. In total, 64 SLE smokers and 128 SLE nonsmokers were included in the case-control analysis. Compared to nonsmokers, there was more nephropathy (59.4% vs.39.8%), proteinuria (54.7% vs. 35.2%), and photosensitivity (35.9% vs. 18%) found in smokers (p<0.05). There was no significant difference in autoantibody production between smokers and nonsmokers, although smokers tended to show more anti-dsDNA positivity (p = 0.358). Smokers also tended to have more active disease (SLEDAI scores: 12.58 ± 8.89 vs.10.5 ± 7.09), but these differences were not significant (p = 0.081; Tables 3 and 4).

**Table 3:** Comparison of clinical manifestations between smokers and nonsmoker systemic lupus erythematosus patients in the case-control study.

	smokers(N = 64)	Nonsmokers(N = 128)	P value
Fever	21.9	24.2	0.718
Rash	43.8	32.8	0.138
Alopecia	26.6	24.2	0.724
Photosensitivity	35.9	18.0	0.006
Oral ulcers	18.8	14.1	0.399
Arthritis	46.9	56.3	0.226
Myositis	6.3	3.1	0.444
Pleuritis	17.2	10.9	0.225
Pericarditis	12.5	10.9	0.749
Vasculitis	12.5	9.4	0.504
Nephropathy	59.4	39.8	*0*.*011*
Renal cast	3.1	4.7	0.721
Microscopic hematuria	31.3	20.3	*0*.*094*
Proteinuria	54.7	35.2	*0*.*010*
Sterile pyuria	3.1	3.1	1.000
Nervous system	7.8	3.9	0.305
Hematological abnormalities	59.4	57.8	0.836

**Table 4:** Comparison of laboratory findings and SLEDAI scores between smokers and nonsmoker SLE patients in the case-control study.

	smokers(N = 64)	nonsmokers(N = 128)	P value
Hypocomplementemia	60.9	70.3	0.192
ANA positivity	85.3	88.3	0.115
Anti dsDNA antibody positivity	56.3	49.2	0.358
Anti Sm antibody positivity	10.9	12.5	0.753
Anti RNP antibody positivity	17.2	11.7	0.296
Anti SSA antibody positivity	23.4	21.1	0.711
Anti SSB antibody positivity	17.2	10.9	0.225
Anti rRNP antibody positivity	26.9	23.3	0.722
APL antibody positivity	42.3	40.7	0.894
SLEDAI score	12.58±8.89	10.5±7.09	*0*.*081*

SLE, systemic lupus erythematosus; SLEDAI, SLE disease activity index; ANA, antinuclear antibody; Sm, Smith; RNP, ribonucleoprotein; rRNP, ribosomal RNP; APL antiphospholipid.

## Discussion

It is well-known that cigarette smoking is harmful to human health and increases the risk of pulmonary carcinoma. However, the relationship between smoking and SLE is still controversial. Previous studies have mostly focused on the risk of developing SLE. Ghaussy*et al*. reported a markedly higher odd’s ratio (OR) than that of other studies in a predominantly Hispanic population: 6.7 for current smokers and 3.7 for ever smokers, whereas others reported ORs ranging from 0.9 to 3.06 for current smokers and 0.6 to 1.2 for ever smokers [6,9,12–16]. Two studies showed that higher daily or cumulative exposure to cigarette smoking was associated with a higher risk of SLE [14,16], suggesting that smoking status may confer an immediate risk for SLE onset, while cessation of smoking decreased this risk. On the other hand, two large prospective cohort studies failed to observe an association between cigarette smoking and development of SLE [17,18]. Similarly, Simard *et al*., found that exposure to cigarette smoke in early life did not increase the risk of development of SLE in adult women [19,20]. However, few studies have investigated the relationship between cigarette smoking and clinical manifestations in SLE patients other than those involving the skin.

Following adjustments for age and gender, our study showed that photosensitivity was more frequent in smokers. This finding is consistent with a study by Bourré-Tessier *et al*. [21], which also showed that current smoking was associated with active SLE rash, and ever smoking was associated with discoid rashes, as well as the American College of Rheumatology total cutaneous score. Similar results were found in other studies [22–24], which reported that smoking was a risk factor for development of cutaneous lupus and associated with the severity of cutaneous lesions. However, our study did not show an association between smoking and rashes. This may be due to the unavailability of differential diagnose of cutaneous diseases from CSTAR. Of particular note, past history of smoking was not associated with active skin rash raised the potential of a reversible influence: cessation of smoking may decrease active skin disease [21]. Unfortunately, our study was unable to further dissect this hypothesis with available data due to the limited number of subjects. In addition, mechanisms underlying the relationship between smoking and active SLE rash are still unknown, with some reports suggesting that smoking may decrease the effectiveness of antimalarial agents and exacerbate skin lesions [25]. In contrast, other studies have not observed an association between smoking and antimalarials[21,26].

Lupus nephritis is a common and severe complication that worsens the prognosis in SLE patients, especially if proteinuria is present. We observed that SLE patients who smoke had more nephropathy and proteinuria. However, in a separate study, the incidence of lupus nephritis itself was not associated with smoke exposure [27]. Till now, the relationship between smoking and renal disease has been investigated mostly in the general population. Cigarette smoking is an independent risk factor for progression of established chronic kidney disease, as well as development of hyperfiltration and proteinuria in the general population, especially men [28–34]. Smoking cessation could reduce the rate of decline inglomerular filtration rate and increase dialysis-free survival in patients with progressive kidney disease [35]. In addition, Ward *et al*. observed that among patients with lupus nephritis, those who smoked had accelerated development of end-stage renal disease [1]. The median time to end-stage renal disease among smokers was 145 months and among nonsmokers was greater than 273 months. In summary, smoking appeared to be an important, potentially modifiable factor influencing the prognosis of patients with lupus nephritis [1].

Other clinical manifestations have been reported to be associated with smoking in SLE patients. Rubin *et al*. reported that current smokers had more episodes of pleuritis, peritonitis, and neuropsychiatric symptoms than former or nonsmoker SLE patients [36]. A study by Moraes-Fontes*et al*. showed neuropsychiatric SLE was associated with a higher frequency of smoking (78%) compared to the non-neuropsychiatric SLE group (26%) [37]. In our study, no differences were found in the above manifestations between smokers and nonsmokers. In addition, smoking has also been associated with a higher instance of thrombotic events and vascular necrosis among SLE patients [38–40]. Some studies have shown that cigarette smoking has an independent effect on cardiovascular disease and is the only significant risk factor for peripheral vascular disease in SLE patients [41,42].

Autoantibodies are one of the characteristic features of SLE. In the present study, we did not find any association between smoking status and all autoantibodies examined in our cohort. Previous studies have mostly focused on anti-dsDNA, an antibody known to correlate with disease activity in SLE. Different results have been reported in previous studies. In a retrospective case-control analysis, Freemer*et al*. found a positive association between current smoking status and the presence of anti-dsDNA antibodies (OR = 4.0; 95% confidence interval: 1.6–10.4). Former smokers, however, were not found to be at increased risk for these antibodies compared to nonsmokers [7]. Preliminary analysis of smaller groups of Hispanic, Asian, and African American patients with SLE from the same cohort were also performed. Although not statistically significant, current smoking was associated with dsDNA seropositivity in Hispanic patients with SLE (OR = 5.02; 95% confidence interval: 0.19 to 134). No such associations were found among African American or Asian patients [7]. However, an earlier study reported a negative association between smoking and immunoglobulin G anti-DNA autoantibodies, both in human and murine SLE [36]. In this study, a negative correlation between smoking and immunoglobulin G anti-dsDNA antibodies in newly diagnosed SLE patients was reported and, in accordance with the mouse model, patients who discontinued smoking prior to diagnosis had higher levels of dsDNA antibodies than current and nonsmokers. Another study reported similar findings, with current smokers having significantly lower levels of anti-dsDNA antibodies than ex-and nonsmokers [4]. These findings suggest that humoral immune suppression may occur with the initiation of smoking, but a rebound effect with increased levels of these antibodies may manifest after cessation of smoking. Results from Young *et al*. were consistent with our finding that smoking is not associated with autoantibody production in SLE patients [43]. It is likely that differences in genetics and race, different autoantibody detection methods, and the attenuation of anti-dsDNA titers by pharmacological treatment may serve to explain the reported discrepancies. On the other hand, Young *et al*. also observed a decreased rate of anti-nRNP68 antibody seropositivity and increased rate of anti-nRNP A seropositivity in SLE patients that smoke [43].

Similar to anti-dsDNA results, the association between smoking and SLEDAI scores has been controversial in previous studies. In our study, we observed higher SLEDAI scores in smokers. Although adjusting for age and gender showed that this difference was not significant (p = 0.081), smokers still had higher SLEDAI scores than nonsmokers (12.58 ± 8.89 versus 10.5 ± 7.09, respectively). Likewise, Ghaussy*et al*. also found that SLEDAI scores were significantly higher in current smokers compared to former and nonsmokers in their retrospective study [2]. This is consistent with the increased anti-dsDNA positivity found by Freemer and colleagues. Some have suggested that a biological mechanism linking smoking and SLE is the association between current smoking behavior and the presence of anti-dsDNA antibodies. This would also explain the epidemiologic association of active cigarette use with increased severity of SLE reported previously [8]. On the other hand, unlike the common phenomenon that severe SLE disease activity is often associated with a low serum complementation, we found less hypocomplementemia (60% vs. 69.5%) in smokers compared with non-smokers, though not significant. The reason maybe was that both microscopic hematuria and proteinuria were 4 scores each and hypocomplementemia was only 1 score in SLEDAI score, hypocomplementemia had less influence on disease activity compared with microscopic hematuria and proteinuria. Thus, it is accessible that a little decreased frequency of hypocomplementemia was found in smokers even though they had greater disease severity. In contrast, other studies did not find significant associations between smoking and SLEDAI scores [3,4,44].

In summary, cigarette smoking may be a trigger for the development and worsening of SLE, especially with respect to renal damage. Our findings suggest that smoking cessation should be particularly encouraged in Chinese patients with SLE.

## Supporting Information

S1 Table Primary data of smokers in SLE patients. Demographic data and clinical manifestations are included. Systemic involvement was measuredby SLE classification criteria, which includedmalar rash, discoid lesion, photosensitivity, oralulcers, arthritis, serositis, hematologic involvement,nephropathy, and neurologic involvement.
(PDF)
