# Supplementary material for: Chinese Systemic Lupus Erythematosus Treatment and Research Group Registry VI: Effect of Cigarette Smoking on the Clinical Phenotype of Chinese Patients with Systemic Lupus Erythematosus
Source: PLoS One. 2015 Aug 17;10(8):e0134451. doi: 10.1371/journal.pone.0134451 (PMC4539270; doi:10.1371/journal.pone.0134451)
Supplement: S1 Table — Demographic data and clinical manifestations are included. Systemic involvement was measuredby SLE classification criteria, which includedmalar rash, discoid lesion, photosensitivity, oralulcers, arthritis, serositis, hematologic involvement,nephropathy, and neurologic involvement. (PDF) [file pone.0134451.s001.pdf]

S1 Table：Primary data of smokers in SLE patients

No.	gender	Age(yrs)	disease duration (months)	Fever	Rash	Alopecia	Oral ulcers	Photosensitivity	Arthritis	Myositis	Pleuritis	Pericarditis	Vasculitis	Nephropathy	Renal cast	Microscopic hematuria	Proteinuria	Sterile pyuria	Nervous system	Hematological abnormalities
1	M	22.4	13.3		+	+							+	+			+			+
2	M	34.0	31.6				+	+	+					+			+			
3	M	29.4	4.8	+										+		+	+			+
4	M	50.3	50.5											+		+	+			+
5	M	25.5	18.7		+			+	+											
6	M	19.3	49.3	+	+			+	+											+
7	F	56.7	198.0		+	+	+	+						+			+			+
8	M	52.3	5.1								+			+	+		+			
9	F	32.5	14.0	+	+		+					+								+
10	F	29.9	40.6					+												+
11	M	43.4	63.1	+	+	+		+											+	
12	M	35.6	209.9		+	+			+											
13	M	63.8	2.3		+				+					+			+			
14	M	28.6	31.5					+	+		+			+		+	+			+
15	M	52.5	193.5						+										+	+
16	F	59.0	12.0			+	+	+	+				+	+			+			
17	F	36.3	94.5						+	+										
18	M	27.2	10.6	+	+	+					+	+	+	+		+	+	+		+
19	M	59.3	4.5		+	+		+					+	+		+	+			+
20	M	45.5	1.9	+			+		+		+			+		+	+		+	+

21	M	25.6	12.7		+				+					+			+			+
22	M	66.1	13.4	+	+				+		+	+								+
23	M	24.8	7.0		+			+					+							+
24	M	19.4	1.8	+		+														+
25	M	35.6	135.9						+	+				+		+				+
26	M	47.9	1.6	+					+		+			+			+			
27	M	36.3	25.1	+		+	+													+
28	M	44.7	70.1	+	+				+					+		+	+		+	+
29	M	61.1	6.8		+							+		+		+				+
30	M	52.9	68.9					+						+		+	+			+
31	M	37.7	43.0											+			+			+
32	M	28.2	21.6																+	+
33	F	48.0	133.3						+		+			+			+			+
34	M	40.1	8.9		+	+	+													
35	M	43.2	49.2		+			+												+
36	M	38.3	2.7						+					+		+	+			+
37	M	77.5	39.9						+											
38	F	17.2	16.8					+						+		+	+			
39	F	43.1	95.9					+	+											+
40	M	36.7	9.6	+	+	+	+	+	+					+	+	+	+			
41	M	38.0	95.1		+	+	+	+		+										
42	M	61.6	32.5		+	+		+					+	+		+	+			
43	M	49.7	19.7	+	+	+		+						+			+			
44	M	57.8	7.5		+	+		+												
45	M	32.1	90.6		+				+											

46	M	25.2	15.6				+				+	+								
47	M	54.9	8.0		+				+											+
48	M	23.0	32.5											+		+	+			+
49	M	26.1	68.2						+					+		+	+			+
50	M	36.6	94.6											+		+	+			
51	M	63.7	12.4								+			+		+	+			+
52	M	54.1	13.1								+			+		+	+			+
53	M	49.2	160.2					+	+					+			+			+
54	M	28.2	--						+											
55	F	40.8	3.8		+	+	+			+		+	+	+			+			+
56	F	31.5	20.9				+		+			+	+	+			+			
57	F	45.6	1.3		+			+						+			+			
58	F	26.5	2.3	+		+					+	+		+			+			
59	F	33.2	7.3		+			+						+			+			+
60	F	28.3	2.2		+															
61	F	27.8	2.4						+					+		+		+		+
62	F	78.7	18.3						+					+			+			
63	F	30.6	114.5					+	+											+
64	F	41.4	38.8						+											+
65	F	52.7	3.9						+											
